# Recognising the importance of chronic lung disease: a consensus statement from the Global Alliance for Chronic Diseases (Lung Diseases group)

**DOI:** 10.1186/s12931-022-02297-y

**Published:** 2023-01-13

**Authors:** Gillian Sandra Gould, John R. Hurst, Antigona Trofor, Jennifer A. Alison, Gregory Fox, Muralidhar M. Kulkarni, Craig E. Wheelock, Marilyn Clarke, Ratika Kumar

**Affiliations:** 1 Faculty of Health, Southern Cross University, Coffs Harbour, Australia; 2 UCL Respiratory, University College London, London, UK; 3 Grigore T. Popa University of Medicine and Pharmacy, Iași, Romania; 4 The University of Sydney, Sydney, Australia; 5 Department of Community Medicine, Kasturba Medical College, Manipal Academy of Higher Education, Manipal, India; 6 Unit of Integrative Metabolomics, Institute of Environmental Medicine, Karolinska Institute, Stockholm, Sweden; 7 Department of Respiratory Medicine and Allergy, Karolinska University Hospital, Stockholm, Sweden

## Abstract

**Background:**

Chronic respiratory diseases are disorders of the airways and other structures of the lung, and include chronic obstructive pulmonary disease (COPD), lung cancer, asthma, bronchiectasis, interstitial lung diseases, occupational lung diseases and pulmonary hypertension. Through this article we take a broad view of chronic lung disease while highlighting (1) the complex interactions of lung diseases with environmental factors (e.g. climate change, smoking and vaping) and multimorbidity and (2) proposed areas to strengthen for better global patient outcomes.

**Conclusion:**

We suggest new directions for the research agenda in high-priority populations and those experiencing health disparities. We call for lung disease to be made a research priority with greater funding allocation globally.

## Introduction

Chronic respiratory disease is one of five non-communicable disease (NCD) areas that contribute to the highest mortality and morbidity globally [1]. Chronic respiratory diseases are diseases of the airways and other structures of the lung, and include chronic obstructive pulmonary disease (COPD), asthma, bronchiectasis, interstitial lung diseases, occupational lung diseases and pulmonary hypertension [2]. The most important and recognised risk factors for lung morbidity include threats such as smoking, indoor, and outdoor air pollution, occupational exposures, and poverty [1]. However, there are several emerging threats and risk factors for chronic lung diseases which include increasing frequency and intensity of climate change events such as bushfires and emerging nicotine delivery systems (e.g., e-cigarettes) and increasing health complexity due to multimorbidity. Chronic lung disease following COVID-19 is also recognised as a major burden to patients and the healthcare system [3]. Disadvantaged populations such as the homeless, Indigenous Peoples, the elderly, migrant populations, and refugees may be disproportionately impacted by these risk factors for lung health. On the basis of this concern, the Global Alliance for Chronic Disease (GACD) announced a call for grants in 2016 focusing on Lung Diseases in Low- and Middle-Income Countries (LMIC), and priority populations in High Income Countries (HIC), and allocated $60 million to this research. Outside of the GACD, the investment has otherwise been labelled inadequate and inequitable; chronic respiratory disease is a poor cousin compared to other NCDs including cancer, cardiovascular disease and diabetes. Lung diseases received just 2% of grants according to the World Health Organisation in their analysis of research expenditures, with respiratory disease being 13th on the list despite chronic lung diseases such as COPD being the 6th leading cause of disability and 5th leading cause of early death [4, 5]. Within lung diseases research, HIC receive the majority of the funding, whereas the burden of disease from chronic lung diseases is disproportionately higher in LMIC. [6]

The GACD Lung Diseases Research Programme has projects operating in 33 countries. The GACD website lists 17 current projects related to lung health compared to 34 projects related to hypertension and mental health and 38 related to diabetes [7]. The Lung Disease projects funded by GACD predominantly focus on prevention of tobacco related harms to the lungs including smoking, second-hand smoke, and e-cigarettes with a small minority of studies focussing on tuberculosis, COPD, and household air pollution.

As a group of lung health representatives from the 16 countries whose major funders collaborate to make the GACD Network, we call for lung health to be elevated up the list of organ-related priorities, especially in the wake of the SARS-CoV-2 (COVID-19) pandemic. The aims of this article are to (a) highlight the complexity and interconnections between major risk factors to lung health (b) suggest new directions for the research agenda in LMIC and HIC where high-priority populations, such as Indigenous peoples and those living in low socio-economic environments, bear the brunt of health disparities.

## Environment effects on lung health

### Climate change

In recent times, climate change has become one of the most discussed phenomena, which has transformative effects on health, economics, ecosystems, and geography. Direct effects of climate change such as severe dust storms, and bushfires or wildfires can adversely affect lung health.

Dust exposure has been linked to Influenza A virus, pulmonary coccidioidomycosis, bacterial pneumonia, and meningococcal meningitis [8]. It has also been associated with NCDs such as COPD, asthma, sarcoidosis, and pulmonary fibrosis [9]. Dust storms can result in increased inhalation of minute particles into the lungs (e.g., PM2.5 and PM10), and can cause and exacerbate symptoms of existing chronic lung disease. Dust inhalation also increases the risk for developing Desert Lung disease or non-occupational silicosis when the lung macrophages mount an inflammatory response to the silica elements of the dust particles that results in lung fibrosis. The lungs can then mount an immune response resulting in fibrosis. Dust inhalation exacerbates the symptoms of chronic obstructive lung disease (COPD) [10]. Dust storms can carry a wide variety of allergens such as pollen, dust mites, fungal spores, and man-made pollutants. The prevalence of asthma is increasing [11], and the increasing frequency of dust storms can be one of the drivers. Desert dust activates a cascade of cytokine mediated immune response resulting in production of IgE, lymphocytes, granulocytes, dendritic cells resulting in increased mucous production and airway remodelling [10]. There is also an increased production of Il-17 that activates neutrophils and facilitate their migration into the areas of lungs where the dust has settled [10].

### Bushfires

Decades of climate change have resulted in an increasing prevalence of wildfires or bushfires [12]. The bushfire crises in Australia during 2019–2020 and recent wildfires in Greece and the US are risks to lung health for those who live in the affected areas. Bushfire smoke contains noxious gases and particulate matter (PM2.5 and PM10 particles, nitric oxides, carbon monoxide (CO) and other toxic gases) which upon entering the lungs and blood stream can result in increased respiratory hospital admissions and use of asthma medication. cardiovascular events, and exacerbations [13]. From data obtained from Sydney, Australia in 2011, an increase in PM2.5 by > 20 µg/m^3^ increased daily all-cause mortality by 5.6%, cardiovascular mortality by 4.5%, and respiratory mortality by 6.1% [14]. Tobacco smoke and bushfires release pollutants into the environment and may have similar ill-effects on lung health. Concurrent smoking and exposure to bushfire smoke is more common in populations with a higher prevalence of tobacco smoking, such as Indigenous People, rural and remote communities, and people who experience mental illness [15]. The combination of tobacco smoking and exposure to bushfire smoke cause intergenerational effects by causing low birth weight in babies born to exposed women [16].

### Indoor biomass burning

A large proportion of populations in low and middle income countries burn biomass such as wood and coal, crop waste and animal dung indoors for cooking, lighting and heating [17]. Women and children are disproportionately affected by the indoor environment saturated with resultant indoor pollution [17]. Studies have shown that persons exposed to biomass indoor pollution can experience decreased and impaired lung growth and development, and an increase in cough and wheeze [18]. Compared to smoking, indoor biomass burning induced COPD is associated with more cough and phlegm, airway thickening and air trapping [19]. Enhanced health education surrounding the harms of indoor air pollution combined with greater access to clean stoves and cleaner fuels may help limit the harms of indoor biomass burning [20].

## Vaping and lung health risks

E-cigarettes first became available in 2003, and have become a relatively new threat to lung health due to their growing usage. Newer devices and flavours make these products more appealing to diverse age groups and populations [21]. E-cigarettes are battery-operated devices, which heat a solution of nicotine and other additives to produce inhalable nicotine vapour. While some experts view them as a tobacco harm reduction method, they are widely considered a potential new threat to lung health. The use of these devices is mired in controversy because of concerns surrounding their benefits in comparison to alternative approaches to smoking cessation, adverse effects, the potential renormalisation of smoking and concerns about uptake by young non-smokers [22]. Research into E-cigarettes is still nascent, relating to their short- and long-term usage safety profile, the gateway effect among young non-smokers and possible decrease of complete nicotine cessation, with dual usage of tobacco and e-cigarettes becoming a concern. There is no consensus among different countries about regulation of e-cigarettes and the laws range from complete ban on e-cigarette usage (India), nicotine liquid being only available on prescription (Australia), to promoting e-cigarettes as a smoking cessation method (UK) marketed directly to the public. Establishing a safety profile for e-cigarettes is difficult—considering not only the lack of historical data but also the plethora of devices and fluids available on the market, and the concealment of their contents. Illicit vaping products may contain contaminants (e.g. vitamin E acetate and THC) which may lead to adverse effects such as the outbreak of EVALI (e-cigarette or vaping product use associated lung injury) [23]. Animal studies have shown that vaping may increase vulnerability to coronavirus infections [24], and have severe neurotoxic [25] and cardiovascular side effects such as endothelial dysfunction, increased oxidative stress, and increased cardiovascular morbidity and mortality [26]. Minor, subclinical effects may be frequent—there is data suggesting detrimental effects on lung function following short term exposure to e-cigarette aerosols possibly due to propylene glycol/glycerol—omnipresent components of vaping fluids. There is also a high potential for accidental poisoning in children, with reported fatalities [27]. Reports have shown increasing numbers of people find it extremely difficult to cease vaping, with little evidence to guide health professionals on what approach would be most effective to achieving this goal [28].

E-cigarettes are a complex and unfolding phenomenon; related decision making would most likely benefit from multidisciplinary research involving the fields of chemistry, clinical medicine, public health, psychology, and social science.

## Multi-morbidities and lung health

Increasingly, chronic lung disease seldom occurs in isolation [29]. Tobacco use along with household and environmental exposures are a very common risk factor for a range of chronic disease such as COPD, cardiovascular disease and many cancers including lung cancer [30]. However, apart from smoking and pollution, the link between chronic respiratory conditions and co-morbid conditions is not fully understood. One possible explanation is chronic systemic inflammation which is present in all chronic respiratory conditions as well as other chronic conditions such as ischaemic heart disease and osteoporosis. Therapy for respiratory disease may also be associated with increased risk of co-morbidity, for example inhaled corticosteroids.

Respiratory conditions are among the most common chronic conditions in patients with multimorbidity [31], but assessing this in disadvantaged populations is difficult because of the under-diagnosis of respiratory disease [32]. In a nationally representative primary care study in Scotland, asthma constituted a prevalence of 6% while 52% of individuals with asthma had one or more additional conditions. Likewise the prevalence of COPD was 3%, and 82% of these patients had two or more additional conditions [33]. Among patients with higher number of morbidities along with respiratory conditions such as asthma it has also been observed that these individuals visit the hospital more frequently for unscheduled emergency, in-patient and out-patient care compared to those without any other condition [34].

Findings from the UK General Practice Research Database (GPRD; now Clinical Practice Research Datalink, CPRD) suggest that newly diagnosed COPD patients are also more likely to be diagnosed with angina, cataracts, bone fractures, pneumonia and respiratory infections within the first year of the COPD diagnosis. Patients with co-morbid COPD and ischaemic heart disease are more likely to be undertreated with beta-blockers despite evidence of safety of this treatment. A link between lung diseases and mental health conditions is an area of concern, potentially mediated by tobacco smoking, as a high proportion of mental health clients smoke [35] and find it difficult to cease [36]. Smokers with mental health conditions are more likely to die from smoking-related conditions than their mental health issues [37, 38].

## Proposed areas to strengthen for improved global lung health

In tackling both old and new threats that we have touched on, we would like to espouse further strengthening in key areas, these include the use of better diagnostics, access to evidence based care, pulmonary rehabilitation, palliative care, attention to ethical issues, use of new technologies, and increased dedicated funding for lung disease research and rapid and equitable implementation of effective measures into practice especially in LMIC and priority populations in high income countries. The latter can be achieved through better implementation of guidelines, but there are challenges to doing this in LMIC. [39]

### Better diagnostics

Effective management of chronic lung disease requires timely, accurate diagnosis yet the availability of key tests such as spirometry remains poor in many settings. Access to diagnostics is a feature of the patient charter for COPD[40] and recently published COPD quality standards [41]. Lack of access to diagnostics is a key barrier to the implementation of guideline-care in LMICs [39].

The challenge is much more than the availability of equipment, and relates to the training and ability of the front line, community based primary care workforce. In a recent population-based screening study of 10,709 people in 3 LMIC sites, the overall prevalence of COPD was 9.4% and > 95% were previously undiagnosed [42]. There is now the need to test implementation of tools to screen for COPD in LMIC settings, and to understand if screening is of benefit to individuals and society.

### Inequities and access to evidence-based care

The growing burden of COPD, particularly in low socio-economic status societies will increase health disparities due to lack of access to evidence-based care. The main determinants of COPD disadvantage relate to discrimination (based on ethnicity, religion, sex, age), physical and mental disabilities, socio-economic status [43]. To alleviate health inequities for people with COPD there should be better case finding, education programs for the general population to raise awareness of COPD and the treatments available, upskilling of health care workers in the management of COPD patients, improved access to cost-effective evidence-based interventions, smoking cessation programs to slow decline in lung function, and environmental initiatives to reduce air pollution and occupational exposures to dusts to improve lung health [44].

### Rehabilitation of chronic lung diseases

Pulmonary rehabilitation, combination of exercise training and education for people with chronic lung disease, has unequivocal evidence of effectiveness in improving health-related quality of life, reducing symptoms of breathlessness and fatigue [45] and reducing hospitalisations [46]. Pulmonary rehabilitation is cited as best-practice management in many national guidelines [47–50]. Such programs have been shown to be one of the most cost-effective treatment strategies for COPD [51].

A key issue is that pulmonary rehabilitation is not widely available. It is estimated that less than 2% of the more than 384 million people world-wide with COPD [52] have access to a pulmonary rehabilitation program [53]. The most disadvantaged are people from LMIC where few pulmonary rehabilitation programs are available. There is growing evidence for the effectiveness of pulmonary rehabilitation in low resource settings, as well as for pulmonary rehabilitation using minimal equipment for exercise training which would enable programs to be implemented in low-income countries [54]. However, this has not yet translated into increased availability of programs. Key to the provision of pulmonary rehabilitation is health professionals trained to deliver this intervention. Strategies to build the capacity of health professionals in LMIC to provide pulmonary rehabilitation need to be developed. In addition, in high income countries, Indigenous populations have very limited availability of culturally appropriate pulmonary rehabilitation programs [55].

It is important that the value of rehabilitation for people with chronic lung disease and those recovering from COVID-19 is recognised and that funding is allocated to supporting current programs and in developing new programs, especially in LMIC.

### Palliative care in chronic lung diseases

As is similar for the under-utilisation of pulmonary rehabilitation, there is low access to and implementation of palliative care for chronic lung diseases, despite its effectiveness [56]. Patients who are dying from COPD experience a progressive decline in health, increasing symptoms and reduced autonomy. However, the majority of patients with advanced COPD are not offered palliative care [57]. National and international guidelines, over the past 20 years, have tried to emphasise its value and adoption into practice [58]. Despite this, even in high income countries, among the general population, there is minimal evidence of palliative care being offered to patients with COPD, compared to those with lung cancer, despite the symptoms being potentially just as distressing [59].

Palliative care for lung diseases in low resource settings is likely to be non-existent or minimal. It is profoundly affected by low access to health services and social and cultural beliefs around sharing a terminal diagnosis with the patient [60]. Palliative care services demand a high degree of networks between multiple disciplines and organisations, and unified standards of ethical care may be missing in LMIC [60]. The infrastructure investment within LMIC may need intensive investment for this to occur, but none-the-less this area needs to be highlighted for future research.

### Ethical issues

Lung health is related to many ethical-legislative sensitive considerations [61]. Clinicians have an ethical duty to ensure patients’ benefit, minimize harm and act in respect to patients’ values and preferences, by building a relationship based on truth, respect, and confidentiality [62]. Airborne infections are a major threat to lung health. Thorough detection of infected/non-infected individuals sharing the same environments should dictate infection control measures aiming to respect equity, social responsibility, non-discrimination and individual liberty. The great Spanish Flu pandemic killed millions of people in 1918–1919 and brought an unmet sanitary crisis to mankind, as there were no available antivirals or vaccines to treat it. TB has significant impacts upon the long-term health of many affected individuals, compounded by its association with poverty and other causes of poor health. COVID-19 global pandemic revealed many ethical-legislative gaps that had to be overcome to save lives, such as novel communication tools, confidentiality emergency disclosure, supplies and treatment accessibility [63].

Similar to other end stage diseases, ethical principles of beneficence and non-maleficence are often in conflict with patient’s autonomy when being treated for end stage lung disease. The best approach is to rely on local ethical guidelines and consensus between medical staff, patient and family. Special ethical-legal consideration must be given to end-life related withdrawal from treatment. As such, many hospitals have ethics committees providing individual support to clinicians, patients, and their families [64].

### Technological interventions

While computers and smart phones have become ubiquitous, many individuals in LMIC still do not have access to these resources and we all have different levels of adoption and usage. Digitalized medicine appears to be a driving force for the improvement of diagnostic, treatment, and progress of research in all medical fields. More than half a century ago, the invention of the cathode ray tube revolutionized radiology [65] and opened countless possibilities of development for imaging in medicine. Since then, the utilization of computers has opened the path to integrating artificial intelligence (AI) in all medical fields, radiology being one of the most advanced areas of application by using deep-learning techniques to detect abnormal scans [66].

E-health can improve access to care for those in rural regions, provided internet access, cellular or a telephone signal is available. Telehealth, while on the fringes for many years, since the advent of the COVID-19 pandemic is now more ubiquitous and here to stay. Technologically enabled learning is a growing field for patient (and clinician) education with the use of computers, smartphones, tablets, and mobile applications. Moreover, technological support can effectively promote patient self-care, medication adherence, and physical activity among those with chronic respiratory conditions [67]. With the increased use of telemedicine, digital interventions need to align with clinical guidance, be context-relevant relevant and appropriately reviewed for quality and impact [67]. The use of telehealth and digital solutions has been utilised across the GACD grant recipients and remains on the agenda as a priority in further funding rounds.

### Funding for chronic lung diseases research, implementation, and research

GACD is a Network that prioritises implementation science, thus we are conscious that in many cases, the evidence-base is available but evidence-based interventions are not being implemented where they are needed the most, or in the most effective way. New calls for research are becoming less disease-focused and open for options about NCD conditions, for example prevention of health risks across the lifespan in 2022. The GACD is an exemplar in funders working together to build implementation solutions for NCDs. However, more needs to be accomplished for chronic lung diseases and its causes.

Beyond the GACD, we would like to call for a greater investment in NCD research, especially in chronic lung diseases, from all funding bodies. In 2022, the International Primary Care Respiratory Group published a Delphi study to set a research agenda for primary care for respiratory diseases. Their priorities, set by a large team from HIC and LMIC, include increasing research into management of chronic cough, COPD, asthma, and smoking cessation, including improving medication adherence and self-management [68]. Multidisciplinary teams and management of co-morbidity was a further focus of need.

Williams et al. on behalf of the Global Health Respiratory Network state, in their letter to the Lancet, that the respiratory research community need to work more effectively “to develop, test, implement, and scale-up the necessary multiple and multisectoral strategies to improve respiratory health” [5]. We join their urgent call to all funders to review their investment in research on respiratory health and take affirmative action, especially to benefit those in low resource settings and high priority and Indigenous populations.

## Conclusion

The GACD Network provides a unique opportunity for lung disease researchers to consider the complexity and interconnections between major risk factors for lung health. We suggest new directions for the research agenda in high-priority populations and those experiencing health disparities. These include a greater attention to: the effects of climate change and bushfires on respiratory health, vaping in addition to tobacco smoking, the complexities from multimorbidity, better diagnostics [69] and areas for improvement in provision of rehabilitation, palliative care, the use of digital technology.

We call for lung health to be elevated up the list of research priorities and receive a greater share of funding relative to burden to enable both new and old threats to be adequately researched, translated and implemented. The World literally cannot afford for the lungs to be left behind in the better research and care for NCDs.

## Data Availability

Not applicable.
